# SCAE—Stacked Convolutional Autoencoder for Fault Diagnosis of a Hydraulic Piston Pump with Limited Data Samples

**DOI:** 10.3390/s24144661

**Published:** 2024-07-18

**Authors:** Oybek Eraliev, Kwang-Hee Lee, Chul-Hee Lee

**Affiliations:** 1 Department of Future Vehicle Engineering, Inha University, 100 Inharo, Mitchuholgu, Incheon 22212, Republic of Korea; 22192319@inha.edu; 2 Department of Mechanical Engineering, Inha University, 100 Inharo, Mitchuholgu, Incheon 22212, Republic of Korea; kh_rhee@inha.ac.kr

**Keywords:** fault diagnosis, hydraulic piston pump, stacked convolutional autoencoder, classification, limited data samples

## Abstract

Deep learning (DL) models require enormous amounts of data to produce reliable diagnosis results. The superiority of DL models over traditional machine learning (ML) methods in terms of feature extraction, feature dimension reduction, and diagnosis performance has been shown in various studies of fault diagnosis systems. However, data acquisition can sometimes be compromised by sensor issues, resulting in limited data samples. In this study, we propose a novel DL model based on a stacked convolutional autoencoder (SCAE) to address the challenge of limited data. The innovation of the SCAE model lies in its ability to enhance gradient information flow and extract richer hierarchical features, leading to superior diagnostic performance even with limited and noisy data samples. This article describes the development of a fault diagnosis method for a hydraulic piston pump using time–frequency visual pattern recognition. The proposed SCAE model has been evaluated on limited data samples of a hydraulic piston pump. The findings of the experiment demonstrate that the suggested approach can achieve excellent diagnostic performance with over 99.5% accuracy. Additionally, the SCAE model has outperformed traditional DL models such as deep neural networks (DNN), standard stacked sparse autoencoders (SSAE), and convolutional neural networks (CNN) in terms of diagnosis performance. Furthermore, the proposed model demonstrates robust performance under noisy data conditions, further highlighting its effectiveness and reliability.

## 1. Introduction

One of the most important parts of a hydraulic system’s power transfer is a hydraulic piston pump. It has been extensively employed in a variety of industries, including aircraft, building equipment, marine apparatuses, and more. The pump’s malfunction might cause downtime or perhaps paralyze the entire production line. From the standpoint of one’s own safety, it could result in terrible catastrophes. As a result, a hydraulic pump’s fault detection is critical to worker safety and safe production, and it has attracted considerable scholarly interest [1,2,3]. Hiddenness and intricacy are the two most glaring aspects of the flaws found in actual implementation. Therefore, it is important and valuable to carry out effective condition monitoring and fault diagnostics of a hydraulic piston pump in order to preserve the functionality of the overall hydraulic system.

Studies on conventional fault diagnostic techniques have focused mostly on mechanism and characteristic frequency analysis [4,5]. The results of the diagnostic procedures could be influenced by past knowledge and professional experience. Machine learning (ML) models have caused the researchers working on machinery fault diagnosis tremendous anxiety with the rise of artificial intelligence [6,7,8]. Traditional ML-based fault diagnosis systems consist of five main processes, including signal acquisition, signal preprocessing, feature extraction, feature selection, and fault detection. It has been demonstrated that the vibration method works well for gathering signals from machinery when it is operating at high speeds. However, extraneous signals from unrelated components frequently obscure the vibration signal [9]. Therefore, procedures in signal preprocessing are needed to filter these undesirable signals. In the last several years, the fault diagnosis process has been reduced to three key processes, including signal acquisition, signal preprocessing, and fault detection with the help of deep learning (DL) models [10], because the processes of feature extraction and selection are automated in numerous hidden layers of DL models. DL models, including convolutional neural networks (CNNs), deep neural networks, (DNNs), deep belief networks (DBNs), and stacked sparse autoencoders (SSAEs) [11] have been commonly employed in fault diagnosis systems.

Although DL has demonstrated that it performs diagnostics better than traditional ML, DL models need big datasets for the learning process to be successful [12]. For instance, Shengnan et al. gave a convolutional neural network (CNN) model a machinery dataset with 6000 samples (4200 training samples and 1800 test samples) to achieve an accurate problem diagnosis result [10]. A deep transfer learning model has been employed with limited data (4000 samples in total) for machinery fault diagnosis by Te Han et al. [13]. An optimized DBN is proposed for fault diagnosis of gearboxes in reference [14]. The main benefit of DL models versus traditional ML models is that performance improves as the size of the dataset increases. Once traditional ML models achieve a particular level of diagnosis performance, increasing dataset size has little impact on their performance. It is challenging to get enough data samples in industrial settings to train DL models. Sensor issues are one element that may contribute to a lack of data samples. Poor data quality is a result of sensor issues, according to Zhang et al. [15]. Furthermore, there are still certain limitations in the existing studies, despite the fact that DL-based intelligent approaches have produced some promising outcomes in fault diagnosis. For example, most of the DL-based research has focused on the intelligent problem diagnosis of gearboxes, motors, rotor systems, and bearings. Studies on pumps, particularly hydraulic piston pumps, are scarce. In addition, there has been very little research on the limited and noisy data, despite the adoption of several evolutionary intelligent algorithms based on DL models for fault diagnosis.

The study presented in this paper suggests a stacked convolutional autoencoder (SCAE) to solve the aforementioned issues. Within the proposed SCAE architecture, the gradient information flow can be maximized, and the deep networks trained effectively by stacking the feature maps of the various convolutional autoencoders (CAEs). Parallel to this, efforts are made to reduce dimensions not only in the width and height directions but also in the depth-wise direction, in order to address potential issues brought on by the increased number of parameters resulting from stacking several AEs. Using experimental data from a hydraulic piston pump, the effectiveness of the suggested SCAE-based fault diagnosis system has been confirmed. The findings of this experimental research confirm that, compared to current methods, the suggested method can significantly increase the diagnosis performance of hydraulic piston pump systems in terms of accuracy and efficiency. By conducting ablation investigations, the effectiveness of the suggested method has been thoroughly confirmed, demonstrating that superior diagnosis performance of over 99.5% with minimal variations may be produced. Finally, the suggested method exhibits stable and reliable diagnosis performance in the presence of sparse or noisy data—problems that are frequently present in real-world settings.

The following is a summary of this paper’s significant contributions:

(1) A novel diagnosis technique for hydraulic piston pump systems based on SCAE is suggested. The flow of gradient information within the networks can be enhanced based on stacking various CAEs. As a result, richer hierarchical features can be obtained, and deep neural network designs can be trained quickly.
(2) In order to investigate the proposed model’s capacity to identify hydraulic piston pump conditions from sparse data, the analysis is fed with a small sample dataset that uses only one data sensor.
(3) The number of parameters in the model can be drastically decreased using the dimension reduction module. As a result, SCAE architectures can be used to efficiently build fault diagnosis models. Better generalization performances follow from this.
(4) Experimental findings show that the suggested strategy can perform more effectively than several other standard approaches. The effectiveness of the suggested strategy is thoroughly examined utilizing various analyses.

The remainder of the paper is structured as follows. The background of the suggested approach is presented in Section 2. The method of the experiment is described in Section 3. The outcome generated by the suggested model is addressed in Section 4. Section 5 concludes by outlining the lesson learned and future work.

## 2. Materials and Methods

### 2.1. Convolutional Autoencoder (CAE)

**Figure 1:**
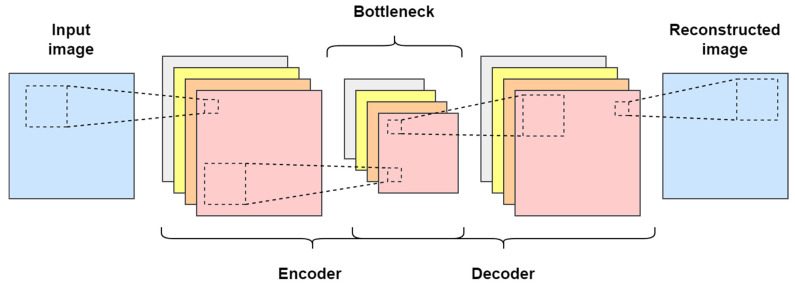
Architecture of traditional convolutional autoencoder (CAE).

A convolutional autoencoder (CAE) is a neural network with convolutional layers that can be used as a feature extractor with images as its input and reconstructs the input at the output. The architecture of the CAE is quite similar to that of traditional autoencoders (AE) [16]. Convolutional layers often contain far fewer parameters than fully connected layers because of the sparse connection and weight sharing, which lowers the danger of overfitting. Therefore, CAE is selected in the proposed model. The two primary components of a CAE’s architecture are the encoding section, which compresses the input to reflect its features, and the decoding part, which reconstructs the input from the compressed form. Convolution and max pooling layers are part of the encoding process, whereas deconvolution and upscaling layers are part of the decoding process [17]. A representation of the CAE architecture is shown in Figure 1.

By using unsupervised learning, CAE can effectively extract the high-efficiency representation of input samples. Its encoder and decoder are written as follows:

(1) $$x_{h}^{i}=f_{en}=S\left(x\cdot W_{1}^{i}+b_{1}\right)$$

(2) $$\dot{x}=f_{de}=S\left(\sum_{i=0}^{N}x_{h}^{i}\cdot W_{2}+b_{2}^{i}\right)$$

where x, $x_{h}^{i}$, and $\dot{x}$ donate row data, the output of the *i*th convolution filter in the encoder, and the reconstructed data, respectively; $W_{1}^{i}$ is the weight of the *i*th convolution filter in the encoder; $W_{2}$ represents the weight of the convolution filter in the decoder; $b_{1}$ and $b_{2}^{i}$ respectively present the biases of encoder and decoder; N denotes the number of convolution filters; and S denotes the nonlinear activation function. The loss function of the autoencoder is expressed as

(3) $$L_{ae}=\sum_{i=1}^{n_{ae}}{\left||x-\dot{x}|\right|}^{2}$$

where $n_{ae}$ represents the total number of unsupervised training samples, which includes data from the source and target domains.

### 2.2. Time–Frequency Transformation

To identify the energy existing in the machinery signal as time and frequency representations, time–frequency analysis is helpful. Machine fault detection has been carried out using a variety of time–frequency transformations, including the spectral kurtosis diagram (kurtogram) [11], empirical mode decomposition (EMD), short-time Fourier transform (STFT) [18], and wavelet transform (WT) [19]. Among these time–frequency transformation methods, STFT is one of the most-used methods for row vibration signals. The STFT transforms a time–domain signal into a time–frequency-domain signal. The long time–domain signal is divided into several pieces using the same size window function, and the fast Fourier transform coefficients (FFT) in the pieces are determined. The mathematical expression of STFT is as follows:

(4) $$STFT\left(\tau ,w\right)=\int_{-\infty }^{\infty }s\left(t\right)w(t-\tau )e^{-j\omega t}dt$$

where $s\left(t\right)$ represents a time domain signal, w(t) represents a window function, t denotes time, and τ denotes time index. Various windowing functions, including triangular, Hamming, rectangular, Kaiser, Gaussian, Blackman, and Hann functions, are available for STFT. The Hann windowing function is typically a good option and is widely used with random data, due to its moderate effect on the frequency resolution and amplitude accuracy of the resulting frequency spectrum compared to other windowing functions [20]. Consequently, the Hann windowing function, which can be expressed in Equation (5), is utilized in this investigation.

(5) $$h\left(t\right)=\left\{\begin{array}{c} 0.5\left[1-\cos\left(\frac{2\pi n}{M-1}\right)\right],0\leq n\leq M-1\\ 0,otherwise \end{array}\right.$$

where n represents time index and M represents the number of samples.

## 3. SCAE-Based Intelligent Fault Diagnosis

**Figure 2:**
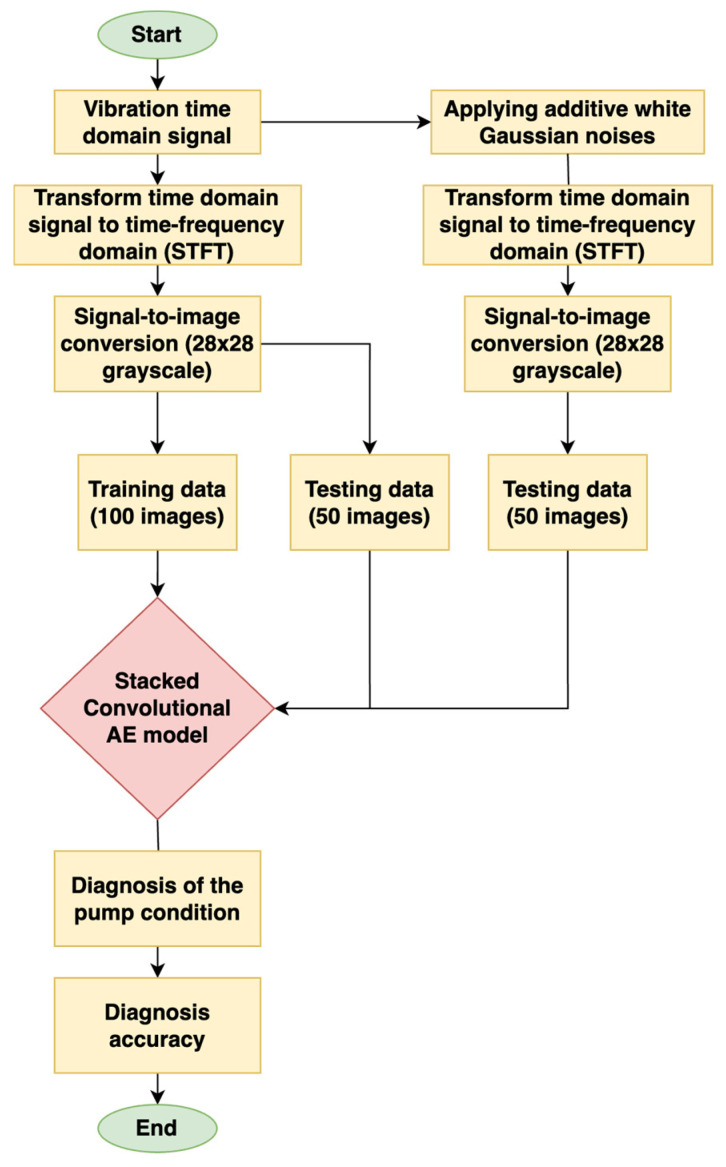
Overall flowchart of the proposed fault diagnosis system.

The overall flowchart of the fault diagnosis method is described in Figure 2. The following seven steps make up the implementation of the proposed fault diagnosis method:

- Step 1: Collect a time domain vibration signal from both normal (healthy) and multiple fault operation conditions.
- Step 2: Transform the time domain signal into a time–frequency domain signal using the STFT method.
- Step 3: Convert the spectrograms of the outputs of STFT to grayscale images (28 × 28).
- Step 4: Split the dataset into a training set and a testing set.
- Step 5: Send the training set and testing set to the proposed SCAE classifier for evaluation of the classifier.
- Step 6: Diagnose the performance of the hydraulic piston pump.
- Step 7: Apply additive white Gaussian noises to the time domain vibration signal, and repeat Step 2 and Step 3 for evaluating the trained SCAE under noisy data samples.

### Stacked Convolutional Autoencoder (SCAE)

**Figure 3:**
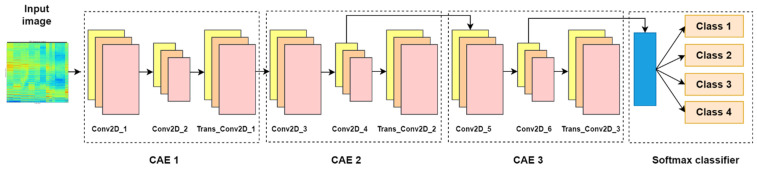
Architecture of the proposed SCAE.

The stacked convolutional autoencoder is developed by stacking three convolutional autoencoders as illustrated in Figure 3. The first CAE of the SCAE reconstructs the input image and can help to protect the model from the noisy data that can seriously influence the overall diagnosis performance of the model. Reconstructed data, which is the output of the first CAE, goes as an input to the second CAE. The features are mapped with the help of 128 filters (filter size 3 × 3) in the encoder of the second SCAE and the mapped features are transferred to the bottleneck for compressing the data. It should be noted here that a large number of trainable parameters could make training less effective and raise the risk of overfitting issues in the networks [21,22]. Hence, not only dimension reduction in width and height with the help of a spatial pooling operation, but also depth-wise pooling with the help of 64 convolutional filters with a size of 1 × 1, is adopted in the bottleneck. These types of dimension reduction modules can greatly reduce the number of trainable parameters. As a result, much deeper neural network designs can be trained quickly and effectively without running into the overfitting problem that the increasing number of parameters may otherwise bring about. Following that, the output of the bottleneck of the second CAE is connected as an input to the last CAE and a similar process in the second CAE is repeated, but the number of filters is different: 64 filters (3 × 3) for the encoder and 32 filters (1 × 1) for the bottleneck. Finally, the bottleneck of the last CAE is connected to a softmax layer. The softmax classifier, which is the last layer of the SCAE network, attempts to classify the features that the CAE processed. The mathematical expression of the softmax is as follows:

(6) $$h_{\theta }\left(x^{i}\right)=\left[\begin{array}{c} \begin{array}{c} p(y^{i}=1|x^{i};\theta )\\ p(y^{i}=2|x^{i};\theta )\\ \vdots \end{array}\\ p(y^{i}=k|x^{i};\theta ) \end{array}\right]=\frac{1}{\sum_{l=1}^{k}e^{\theta_{j}^{T}x^{i}}}\left[\begin{array}{c} \begin{array}{c} e^{\theta_{1}^{T}x^{i}}\\ e^{\theta_{2}^{T}x^{i}}\\ \vdots \end{array}\\ e^{\theta_{k}^{T}x^{i}} \end{array}\right]$$

where k is the number of classes, $\theta_{1}$, $\theta_{2}$, …, $\theta_{k}$ denote the model parameters, and p denotes the probability distribution.

The hyperparameters for the proposed SCAE are selected manually and are shown in Table 1.

The SCAE model proposed in this study introduces several key innovations designed to enhance fault diagnosis performance in hydraulic piston pumps. While the SCAE might appear as a simple stack of three CAEs, it incorporates novel elements that significantly improve its efficacy and robustness, particularly when dealing with limited and noisy data. For instance, the SCAE architecture is not a mere linear stack of CAEs. Each CAE layer is carefully designed and integrated to optimize gradient flow and feature extraction. By meticulously stacking and connecting the CAEs, it is ensured that the model retains maximum information across layers and minimizes the risk of vanishing gradients. This architectural choice is crucial for improving the overall performance and stability of the model during training. In addition, the proposed model features a novel dimensionality reduction module that operates on spatial dimensions (width and height) and the depth-wise dimension. This approach significantly reduces the number of trainable parameters, addressing overfitting and underfitting issues more effectively than traditional stacking methods. The dimensionality reduction module also acts as a form of regularization, ensuring that the model generalizes well to unseen data. The SCAE model’s architecture and dimensionality reduction strategies collectively contribute to its enhanced performance in fault diagnosis tasks. These innovations distinguish the proposed approach from traditional CAE stacking methods and highlight the unique contributions of this study.

## 4. Experimental Data Collection and Preprocessing

### 4.1. Data Description

**Figure 4:**
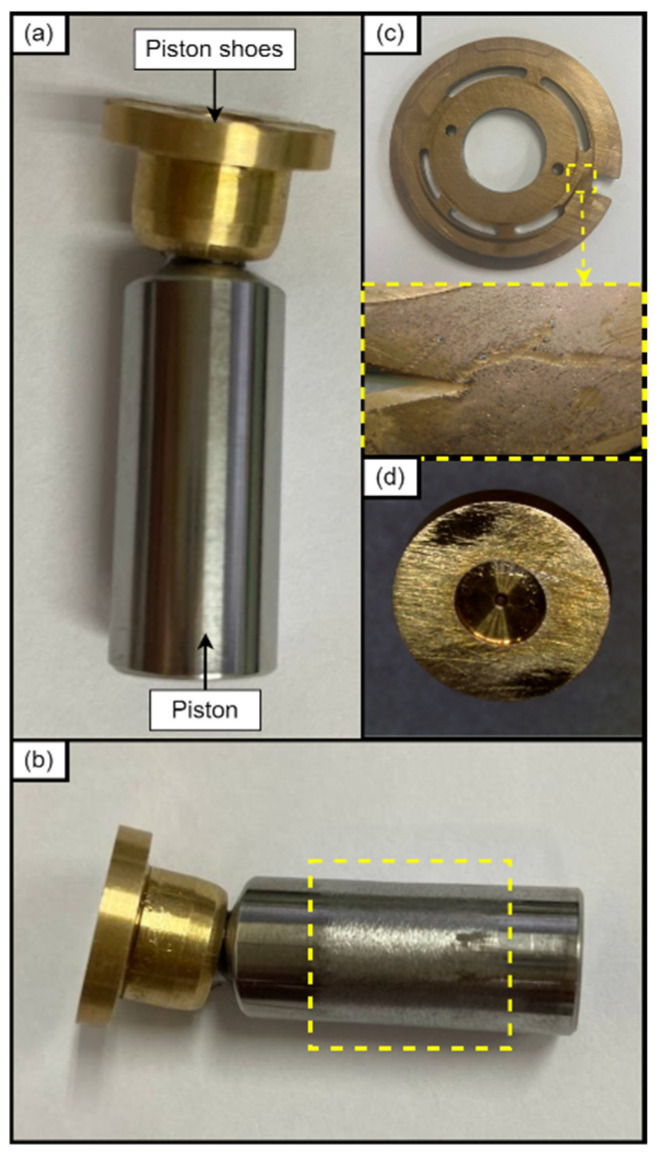
Damaged components of the hydraulic piston pump: (**a**) normal piston, (**b**) worn piston, (**c**) worn port plate and (**d**) worn piston shoes.

Axial piston pumps of the swash plate type are made up of a number of parts, including a driveshaft, swash plate, piston shoes, pistons, valve plate, and housing. An axial piston pump needs to include a port for external drainage because appropriate lubrication is crucial and because improper lubrication causes constant leakage. Axial piston pumps have multiple parts in sliding contact because of their design, which makes them vulnerable to leaks and wear. Because of this, wear is a frequent cause of failure and may be seen in the friction pairs—components in sliding contact—between the piston and cylinder wall and the valve plate and cylinder block. According to studies on the subject, wear accounts for 80% of axial piston pump failures [23]. Furthermore, abrasive wear is the most frequent failure mode, and 38% of aviation failures caused by failed piston pumps are due to it. This failure can be attributed to fluid contamination, wear on various pump components, cavitation, and inadequate lubrication. Increased total leakages, a decline in volumetric efficiency, abnormal vibrations, and abnormal noise are some of the repercussions of the failure mode [24]. Therefore, this study focused on the failure modes related to wear. Three common failure modes, including worn port plate, worn pistons, and worn piston shoes were chosen, as shown in Figure 4.

**Figure 5:**
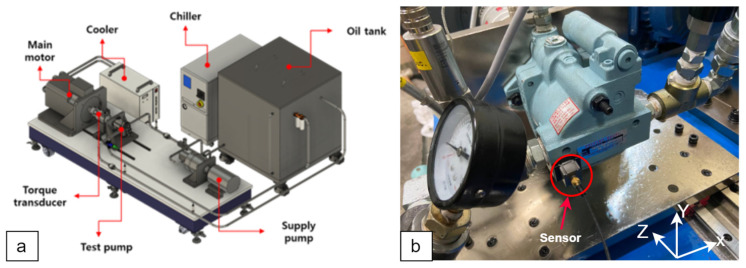
(**a**) Experimental setup. (**b**) The sensor position on the pump.

The experiments were conducted in a test setup of the hydraulic axial piston pump. Specifications of the pump are listed in Table 2. Images of the experimental setup (a) and the tested pump (b) are illustrated in Figure 5. A three-axis accelerometer sensor is installed on the case of the pump to measure the vibration signal. The sensor measures three row vibration signals along the X, Y, and Z axes, as indicated in Figure 5b.

Experiments were conducted in both healthy and faulty conditions to explore the methodology. The defective ones were obtained by putting worn-out and damaged parts into the pump. At a constant pump displacement of 8.0 cm^3^/rev, which corresponds to a swash plate angle of 12.8 degree all conditions were tested under various delivery pressures and angular speed values of the pump, as detailed in Table 3. The measurements of the sensor were recorded on a PC with a DAQ (NI cDAQ-9174) module with a 10 kHz sampling rate and 15-s time duration. The experiments were repeated five times for each operating condition in order to collect different and complex data for analysis of the proposed classifier.

### 4.2. Data Preprocessing

**Figure 6:**
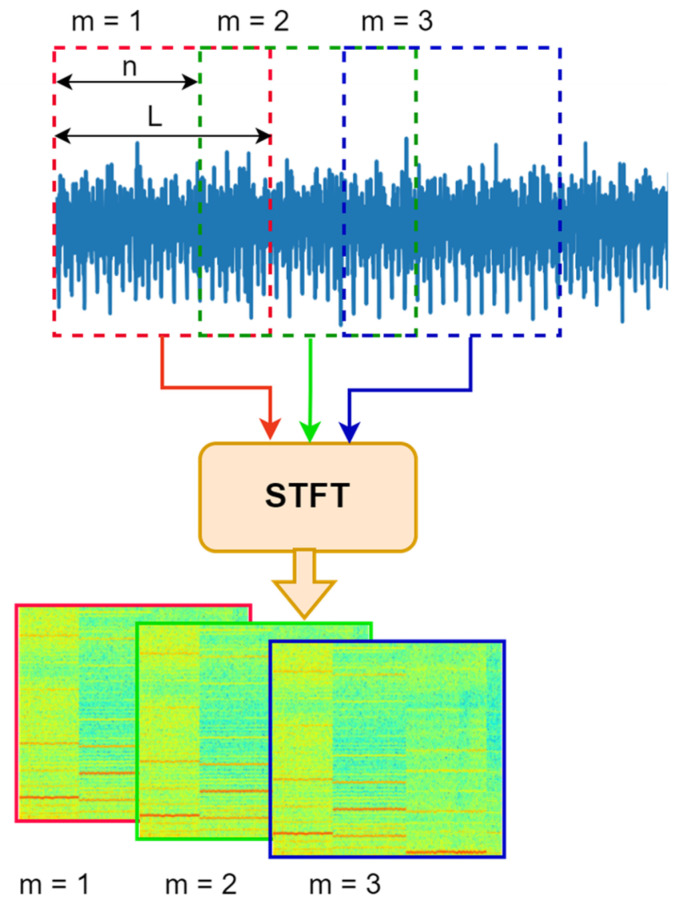
Preprocessing of the time domain signal.

**Figure 7:**
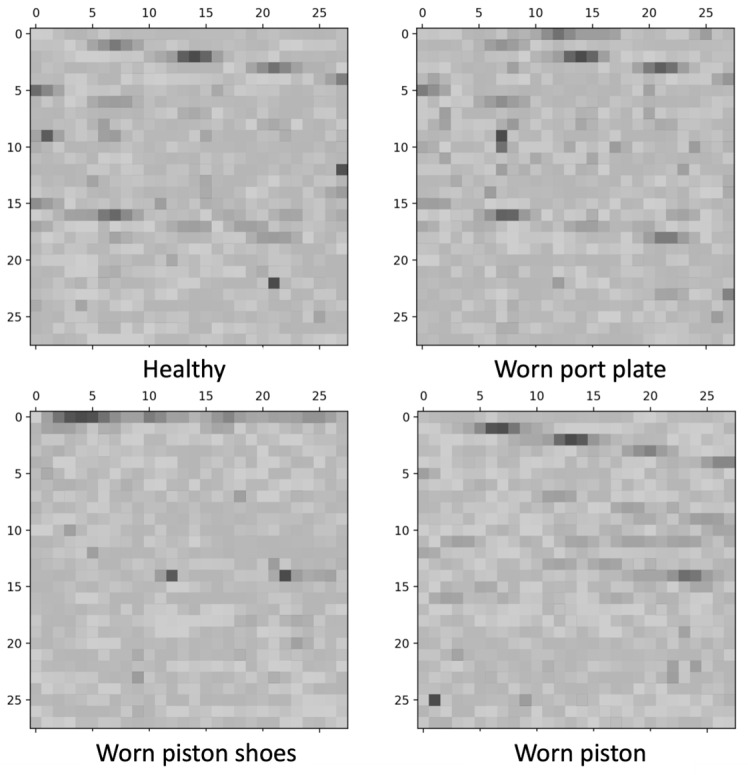
Examples of the generated images for all healthy and unhealthy conditions.

It is required to convert the resulting 1D time series into 2D pictures because the CNN requires 2D input data [25]. For the signal preprocessing, the STFT method is conducted as shown in Figure 6. From the figure, L represents the length of the window, which contains 1600 data points, and n represents the hop length or sliding length of the window, which contains 1024 data points. For each window, 784 fast Fourier transform (FFT) coefficients are calculated. Following that, the output spectrogram of the STFT for each window is converted to a 2D 28 × 28 grayscale image and saved. The examples of the generated images, which correspond to the condition, of angular speed of 1000 rev/min and delivery pressure of 100 bar, for all the states of the pump, are shown in Figure 7.

For the fault diagnosis, each pump condition is assigned a class as shown in Table 4. For each class, 100 images and 50 images are selected randomly from the generated dataset for the training dataset and test dataset, respectively.

## 5. Results and Discussion

In this research, the proposed model was evaluated using a single-sensor system rather than a multi-sensor system. An accurate result might be obtained by the use of a multi-sensor system, but in practical applications, not every sensor will always deliver a good signal for analysis. Therefore, the primary goal was to accurately diagnose faults using small data samples. Furthermore, the effect of the vibration signals that were obtained in three different X, Y, and Z axes on the overall diagnosis performance were investigated.

### 5.1. Traditional Fault Diagnosis

**Figure 8:**
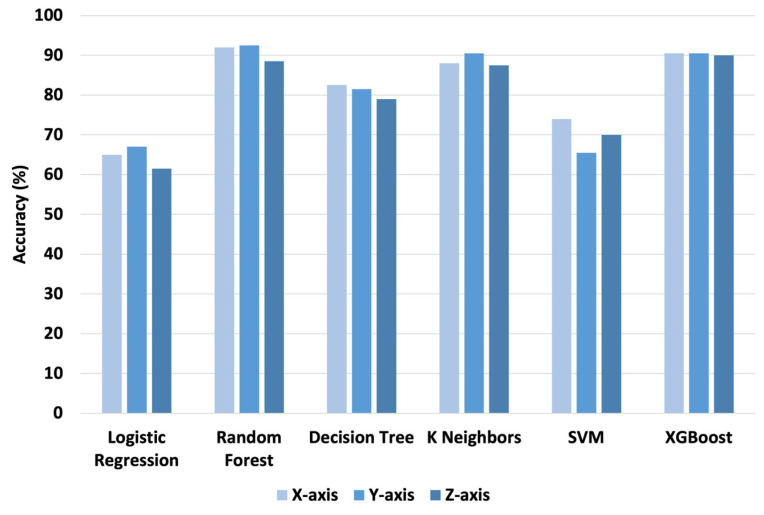
The diagnosis performance results of traditional ML models.

For the analysis, several ML models were used, such as logistic regression (LR), random forest (RF), decision tree (DT), k-nearest neighbor (KNN), support vector machine (SVM), and XGBoost. Thirteen statistical features, as shown in Table 5, were extracted from the time domain vibration signal. The result of the analysis is illustrated in Figure 8. According to Figure 8, there is no significant difference among the models of RF, DT, KNN, and XGBoost in diagnosis performance. These models’ accuracy ranges from around 80% to 92.5%, with the RF model having the best diagnosis performance in the Y axis vibration signal data, while the LR and SVM models show the lowest diagnosis performance, with an accuracy of around 70%.

### 5.2. DL Based Fault Diagnosis

**Figure 9:**
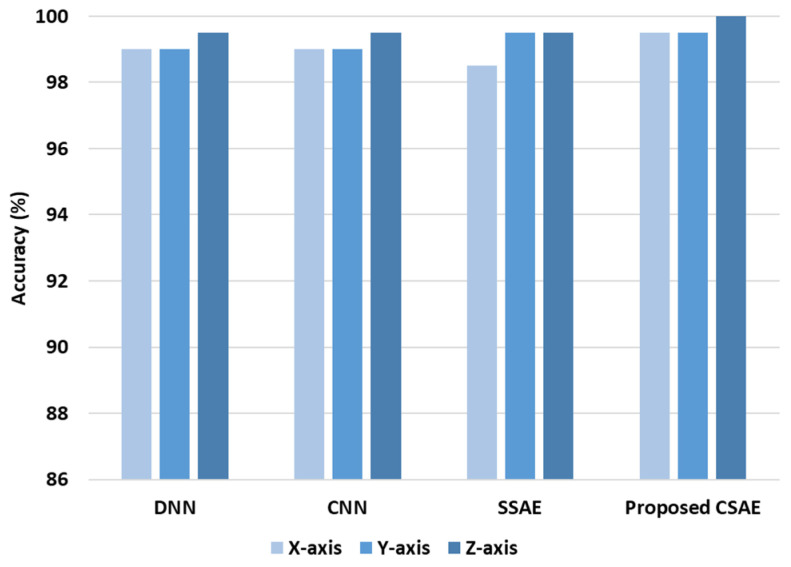
Diagnosis performance of the DL and proposed SCAE models.

In order to conduct a comparative study of models of DL, some popular models such as DNN, SSAE and CNN are chosen. The proposed SCAE and DL models’ diagnosis performance is displayed in Figure 9. There is not significantly difference between the diagnosis performance of DNN and CNN, with the accuracy about 99% for X and Y directions data and about 99.5% for Z direction data. The SSAE achieves 98.5% (for X direction data) and 99.5% (Y and Z direction data), while the proposed SCAE achieves the highest diagnosis performance of 99.5% to 100%. As can be seen from Figure 9, the data of Z direction has more information for diagnosis of the pump, while the rest of the data has not difference. According to the result, since there is no significant difference between the data of the three axes of the sensor, the rest of the analysis has been performed for the data of the X axis.

Dimension reduction is performed in SCAE to reduce the number of parameters required to build effective DL models. The number of trainable parameters for DL-based methods is examined in the research described in this part to confirm the impact of the suggested SCAE-based fault diagnosis approach. The high accuracy of the SCAE model is a result of its superior feature extraction capabilities, effective network architecture, and robust training procedures. These internal mechanisms collectively enable the SCAE model to outperform other DL models in diagnosing faults in hydraulic piston pumps, even under challenging data conditions.

The DNN model has four dense layers. The first two layers have 512 neurons, and the rest of the layers have 256 neurons, and ReLU is used as an activation function. The encoder of the SSAE model consists of three danse layers with 512, 256, and 128 neurons, respectively, while the decoder consists of three danse layers with 128, 256, and 256 neurons, respectively. The bottleneck has 64 neurons, and LeakyReLU is used as an activation function. The hyperparameters of the CNN are detailed in Table 6.

**Figure 10:**
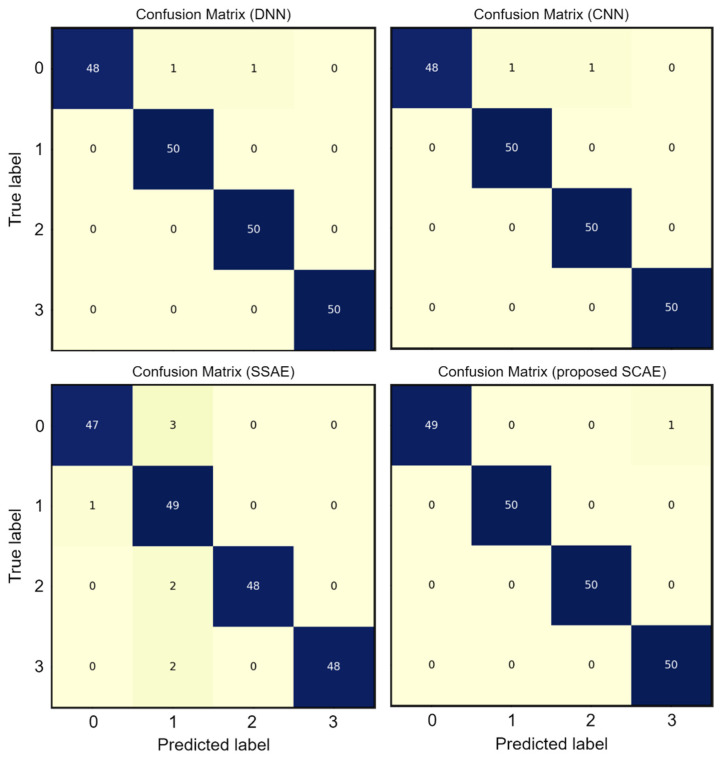
Confusion matrix of the DL-based models.

The confusion matrix is used in order to analyze the classification accuracy of the DL-based models for each fault type. The outcomes are contrasted with those for the proposed SCAE, DNN, SSAE, and CNN. As shown in Figure 10, DNN, CNN, and the proposed SCAE show a classification accuracy of 100% for all fault types, including worn port plate, worn piston, and worn piston shoes, while SSAE shows the worst accuracy. Regarding the healthy condition, DNN and CNN make similar mistakes; meanwhile, the proposed SCAE overcomes the mistakes of the DNN and CNN models. The results prove that the proposed SCAE is superior to the DL-based models and more accurate at classifying all types of pump operating conditions.

**Figure 11:**
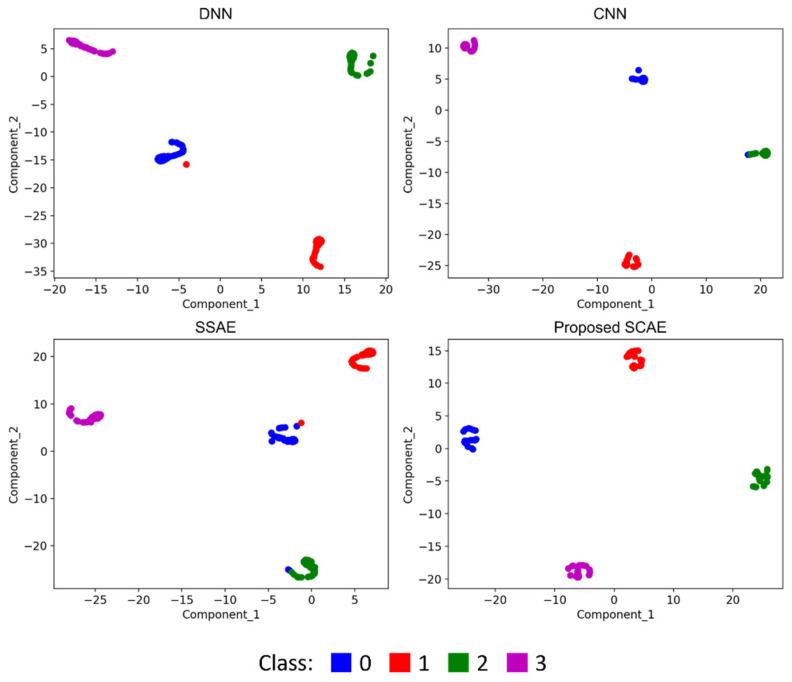
Feature representations of the last layer of DL-based models via t-SNE.

The t-distributed stochastic neighbor embedding (t-SNE) technique, which combines dimensionality reduction and visualization, is a powerful nonlinear algorithm used to represent high-dimensional datasets in a lower-dimensional space. In this study, t-SNE was employed to visualize the feature distribution of the learning results obtained from the output layer of various deep learning models. By representing the feature distributions as probability distributions, we gained insights into the transformation of features throughout the network. Figure 11 illustrates the visualizations generated by t-SNE. From the figure, the extracted features become more distinguishable but there are some overlaps in the DNN, SSAE, and CNN models. Meanwhile, the proposed model demonstrates that after passing through the classifier layer, the features corresponding to each health condition formed distinct clusters, separated by a noticeable distance. These findings indicate that the proposed SCAE successfully utilizes time–frequency images of vibration signals to diagnose faults in hydraulic piston pumps. These visualizations serve as evidence of the effectiveness of the proposed SCAE in fault diagnosis. The clear grouping and separation of features at the final classifier layer demonstrate that the model can accurately classify and differentiate between different fault types in hydraulic pumps based on the time–frequency information extracted from vibration signals. Overall, the t-SNE-based visualization of the output layers of each DL model provides valuable insights into the learned representations and facilitates the interpretation of the network’s performance in capturing and distinguishing fault patterns in hydraulic pumps.

### 5.3. Robustness of Diagnosis Performance under Noisy Conditions

The ultimate objective of creating fault diagnosis methods is to increase the dependability of equipment used in actual industrial settings. Therefore, it is important to take into account potential problems in real-world settings while building fault diagnosis procedures and to create algorithms that are resilient when those problems arise. The quantity of data available can be the first issue in the field. But the proposed SCAE is robust and has accurate results in this area. The second problem that might arise in actual industrial settings is noisy data, which can be brought on by unpredictability in the operating environment and the environment itself. It is impossible to train the diagnosis models to anticipate every possible noise because different noises in the real environment are unavoidably present. As a result, the test data may contain noise that wasn’t present during the training phase, which can significantly reduce the performance of the trained diagnosis models. Hence, it is essential to create diagnosis models that are resilient to different types of noisy data [26,27]. Test datasets are supplemented with additive white Gaussian noises of varying signal-to-noise ratios (SNRs) in order to verify the resilience of the suggested approaches against noisy data. The SNR is defined as follows in decibels (dB):

(7) $${SNR}_{db}={10log}_{10}\left(\frac{P_{signal}}{P_{noise}}\right)=P_{signal,db}-P_{noise,db}$$

where $P_{signal}$ represents the power of the signal and $P_{noise}$ represents the power of the additive white Gaussian noise.

**Figure 12:**
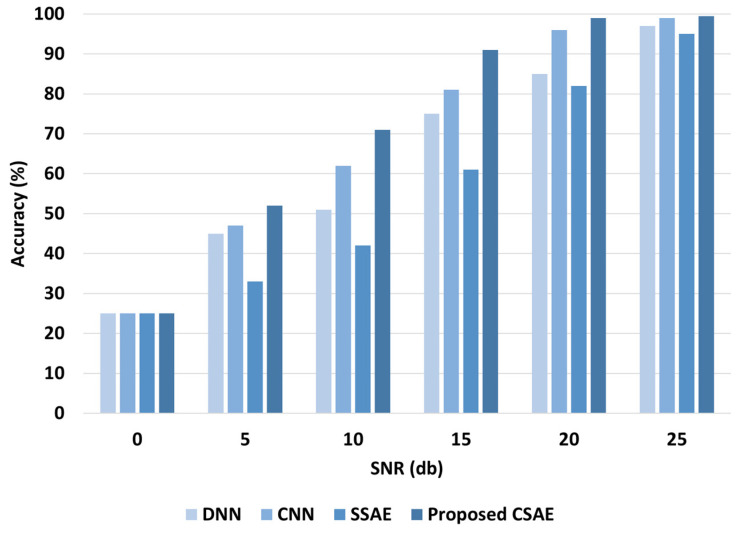
Diagnosis performance of the DL-based models at various additive noise levels.

Figure 12 displays the findings of the diagnostic for the test data with additive noise. It can be seen that for all approaches, the diagnosis performance decreases with increasing noise ratios because the extra noise changes the test data’s distributions and features. Additionally, it can be seen that for all SNR values, the suggested SCAE model performs better than the other DL-based models. Additionally, the suggested model exhibits strong diagnosis performance even when some noise is added, in contrast to the other models, which exhibit very rapid performance loss with additive noise.

In conclusion, the proposed model outperforms previous methods in terms of generalization performance against additive noise. These SCAE benefits could be the cause of this. First, the first CAE of the proposed method plays a key role in denoising the data samples and improving overall diagnosis performance under noisy conditions. Second, better connectivity within network designs enables effective training to produce enhanced features for diagnosis. At the same time, the overfitting issue can be avoided by lowering the number of parameters. As a result, generalization performance is enhanced. Based on the results of the aforementioned experiment, it is confirmed that the proposed SCAE model can work effectively in a variety of situations that may arise in actual operating environments, such as when there is a lack of training data or unavoidable noise conditions. On the other hand, the proposed SCAE model offers a significant advantage in terms of its lightweight architecture. Unlike traditional deep learning models that often require substantial computational resources and extensive memory, the SCAE model is designed to be highly efficient with a reduced number of trainable parameters. This efficiency does not come at the cost of performance. On the contrary, as shown in Table 7, the SCAE model achieves similar or even superior results compared to other models with far fewer parameters. The compactness of the SCAE model makes it particularly well-suited for deployment on embedded systems and edge devices, where computational power and memory capacity are typically limited. This capability opens up new possibilities for real-time fault diagnosis in environments that demand low-latency and resource-efficient solutions. By maintaining high diagnostic accuracy while significantly reducing the computational burden, the SCAE model provides a robust and scalable solution for fault diagnosis applications. This balance between performance and efficiency ensures that the SCAE model can be effectively utilized in practical, real-world settings where traditional, heavier models may not be feasible. The practical implications of the lightweight architecture of the SCAE model are profound. For instance, in industrial applications where real-time monitoring and fault diagnosis are critical, deploying the SCAE model on embedded systems can lead to faster response times, lower operational costs, and enhanced reliability. This makes the SCAE model not only a powerful tool for accurate fault detection but also a practical solution that meets the constraints of modern embedded and edge computing environments. The summary of this study is described in Table 7.

## 6. Conclusions

In this study, a novel SCAE model, three stacked convolutional autoencoders, is proposed for fault diagnosis of a hydraulic axial piston pump. The goal of the SCAE model is that by increasing the gradient information flow and minimizing the number of parameters, it aims to enhance training effectiveness and diagnosis performance. Furthermore, a vibration image generation technique without the need for manual feature extraction is adopted, using the STFT method, in this paper, and the model is tested on the limited data samples. To confirm the effectiveness of the proposed SCAE model, experimental studies are performed and compared with several DL-based models and traditional ML models. The result shows that outstanding diagnosis performance (above 99.5% diagnosis accuracy) and effective training are both possible when utilizing the proposed SCAE. The proposed model has better and faster diagnosis performance when compared to both traditional ML- and DL-based models. In addition, even with additive noise conditions, the suggested model demonstrates steady and resilient performance, proving its superiority for real-world issues.

Future work is required because there are still a number of limitations to be investigated. The efficiency of the suggested model should first be further verified using datasets obtained from various hydraulic piston pumps. Additionally, supervised learning techniques are used to train the majority of fault diagnosis algorithms, including the suggested SCAE model. Sufficient labeled data are also required for the development of these algorithms. It is not always easy to get enough labeled data, though. It is particularly challenging to gather enough fault data and related label information for large-scale, real-world systems. As a result, further study should be done to create approaches for fault identification that make use of unsupervised or transferable learning systems. The proposed SCAE model can be used as the main model in this type of research to improve the performance of the diagnosis. However, the hyperparameter optimization technique can be performed to decrease the number of parameters and increase diagnosis performance as much as possible.

## Figures and Tables

**Table 1:** Hyperparameter settings of the proposed SCAE.

Layers	Number of Kernels (Neurons)	Kernel Size	Activation Function
Conv2D_1 + BatchNor	128	(3 × 3)	LeakyReLU
Conv2D_2 + BatchNor	64	(3 × 3)	LeakyReLU
Trans_Conv2D_1 + BatchNor	128	(3 × 3)	LeakyReLU
Conv2D_3 + BatchNor	128	(3 × 3)	LeakyReLU
Conv2D_4 + BatchNor	64	(1 × 1)	LeakyReLU
MaxPooling2D	-	-	-
Trans_Conv2D_2 + BatchNor	128	(3 × 3)	LeakyReLU
Conv2D_5 + BatchNor	64	(3 × 3)	LeakyReLU
Conv2D_6 + BatchNor	32	(1 × 1)	LeakyReLU
MaxPooling2D	-	-	-
Trans_Conv2D_3 + BatchNor	64	(3 × 3)	LeakyReLU

**Table 2:** Specification of the axial piston pump.

Name	Specification
Model	PV-0B-80-30
Volume/rev	8.0 (cm^3^/rev)
Pressure adjustment range	30.6 to 214 MPa
Permitted peak pressure	25 MPa
Rotating speed	500–2000 1/min
Mass	7.7 kg

**Table 3:** Operating conditions for data acquisition in healthy and faulty states of the pump.

Angular Velocity	Swash Angle	Pressure (Bar)	
		100	200
1000 rev/min	12.8 deg	✓	✓
1500 rev/min	12.8 deg	✓	✓
2000 rev/min	12.8 deg	✓	✓

**Table 4:** Data distribution.

Pump Conditions	Number of Training Data	Number of Testing Data	Class
Normal	100	50	0
Worn port plate	100	50	1
Worn pistons	100	50	2
Worn piston shoes	100	50	3
Total data	400	200	

**Table 5:** Features from the time domain vibration signal.

Parameter	Equation	Parameter	Equation
Mean	$T1=\frac{\sum_{n=1}^{N}s(t)}{N}$	Crest factor Margin	$T8=\frac{T5}{T4}$
Standard deviation	$T2=\sqrt{\frac{\sum_{n=1}^{N}{\left(s\left(t\right)-T1\right)}^{2}}{N-1}}$	Margin factor	$T9=\frac{T5}{T3}$
Variance	$T3={\left(\frac{\sum_{n=1}^{N}\sqrt{\left|s(t)\right|}}{N}\right)}^{2}$	Shape factor	$T10=\frac{T4}{\frac{1}{N}\sum_{n=1}^{N}\left|s(t)\right|}$
RMS	$T4=\sqrt{\frac{\sum_{n=1}^{N}{\left(s(t)\right)}^{2}}{N}}$	Impulse factor	$T11=\frac{T5}{\frac{1}{N}\sum_{n=1}^{N}\left|s(t)\right|}$
Absolute maximum	T5=max⁡|s(t)|	A factor	$T12=\frac{T5}{T2\cdot T3}$
Coefficient of skewness	$T6=\frac{\sum_{n=1}^{N}{\left(s\left(t\right)-T1\right)}^{3}}{(N-1)({T2)}^{3}}$	B factor	$T13=\frac{T7\cdot T8}{T2}$
Kurtosis	$T7=\frac{\sum_{n=1}^{N}{\left(s\left(t\right)-T1\right)}^{4}}{(N-1){\left(T2\right)}^{4}}$		

**Table 6:** Hyperparameter settings of the CNN model.

Layers	Number of Kernels (Neurons)	Kernel Size	Activation Function
Conv_1	16	(3, 3)	ReLU
Conv_2	32	(3, 3)	ReLU
MaxPooling	-	-	-
Conv_3	64	(3, 3)	ReLU
Conv_4	64	(3, 3)	ReLU
MaxPooling	-	-	-
Dense_1	512	-	ReLU
Dence_2	512	-	ReLU

**Table 7:** Summary of the study.

Classifiers	Number of Trainable Parameters	Accuracy on Clean Data (%)	Accuracy on Noisy Data 20 db, (%)
Logistic Regression	-	65	-
Random Forest	-	92	-
Decision Tree	-	82.5	-
KNN	-	88	-
SVM	-	74	-
XGBoost	-	90.5	-
DNN	862,724	99	85
SSAE	749,380	98.5	82
CNN	1,931,076	99	96
Proposed SCAE	391,716	99.5	99

## Data Availability

The data presented in this study are available on request from the corresponding author due to (specify the reason for the restriction).
